# Genetic polymorphism of the prostasin gene in hypertensive pregnant Pakistani females

**DOI:** 10.12669/pjms.37.1.3666

**Published:** 2021

**Authors:** Saima Ejaz, Anwar Ali, Sumaira Riffat, Atif Mahmood, Kamran Azim

**Affiliations:** 1 Saima Ejaz Ph.D. Scholar, Department of Physiology, University of Karachi, Pakistan; 2 Anwar Ali Assistant Professor, Department of Physiology, University of Karachi, Pakistan; 3 Sumaira Riffat (M.Phil.) Lecturer, Department of Physiology, Sindh Medical College, Jinnah Sindh Medical University, Karachi, Pakistan; 4 Atif Mahmood (M.Phil.) Associate Professor, Department of Physiology, Bhitai Medical and Dental College, Mirpur Khas, Pakistan; 5 Kamran Azim (PhD) Professor, Department of Bioscience, Muhammad Ali Jinnah University, Karachi, Pakistan

**Keywords:** Hypertension, Prostasin, Epithelial Sodium Channel

## Abstract

**Objective:**

The study was performed to investigate the association of hypertension in pregnancy with prostasin gene polymorphism in Pakistani females.

**Methods:**

This case-control study was performed at University of Karachi, Pakistan from April 2018 to May 2019. A total of 160 females, including 90 hypertensives and 70 healthy pregnant females, were recruited by purposive sampling after obtaining informed written consent. Genotyping was performed by polymerase chain reaction (PCR) and restriction fragment length polymorphism (RFLP).

**Results:**

The frequencies of the TC and CC genotypes were higher in hypertensive pregnant females compared to healthy controls. A significant difference was evident for CC (P=0.012) genotype; however, no significant difference was observed for TC (P=0.49) and TT genotypes (P=0.06) between control and hypertensive groups. The adjusted odds ratio for CC genotype was 6.2 (P=0.025) and 1.48 (P=0.44) for TC genotype compared to the TT genotype. There was a significantly higher prevalence of the C allele of the prostasin gene at rs12597511 in the hypertensive group, suggesting that this allele is a risk factor for hypertension and cardiovascular diseases.

**Conclusion:**

C allele at rs12597511 of prostasin gene demonstrate as a risk factor for having hypertension in pregnancy.

## INTRODUCTION

Pregnancy hypertension is a significant risk factor for fetal and maternal morbidity and mortality in 10% of pregnancies.^1^ Patients diagnosed with hypertension at 20 weeks of gestation or earlier are labelled as chronic hypertensive whereas-after 20 weeks of gestation, it is called gestational hypertension.^2^ Chronic hypertension is associated with preeclampsia, intrauterine growth restriction, and placental abruption, which eventually leads to serious sometimes life-threatening conditions for both fetus and mothers. Obesity and old age are two major contributors.^3^

Many studies have been performed to identify hypertension susceptibility genes, but few have reported consistent results. Different populations have shown different results on a single genetic variant among non-pregnant females and males. Among those, polymorphisms of certain genes such as β_2_-adrenergic receptor angiotensin-converting enzyme, renin-binding protein, α-adducin, atrial natriuretic factor, angiotensinase C, and the insulin receptor have been linked with the development of hypertension.^4^ However, most of the researchers found a weak association if any or further confirmation is required by others.

Few studies have indicated that prostasin-a serine protease glycosylphosphatidylinositol-anchored (GPI) expressed in distal nephron has a novel mechanism for blood pressure regulation. It regulates blood pressure by activation and proteolytic processing of epithelial sodium channel (ENaC),^5^ which is composed of α, β, and γ subunits in the renal distal tubules. Although, ENaC’s dependent sodium reabsorption accounts for only a small proportion, constitutes a rate-limiting step and involves in the final renal sodium adjustments that ultimately controls blood pressure.^6^ Prostasin is suggested to induce dissolution of an inhibitory peptide from γ-ENaC to activate the cellular channels.^7^ The human prostasin (PRSS8) gene is located at 16p11.2, consisting of five introns and six exons. In the original cloning report, the entire human prostasin gene was sequenced and found to have a 1.2-kb 3’-flanking region, a 1.4-kb 5’-flanking region and a genomic region of 7-kilobase.^8^ In a population of Xinjiang Kazakh, Fang et al. identified the correlation of genetic variants in prostasin gene with essential hypertension, but significant association was observed only with E342K in the hypertensive patients.^9^ Variations in findings from many studies indicate the presence of racial disparities in prostasin polymorphism and suggest the need for further research in other ethnicities with a larger sample size. No study to date has explored the role of prostasin gene mutation in Pakistani hypertensive patients.

This study evaluates the genetic variations of the prostasin gene in pregnant females that exhibit hypertension. In order to gain an insight into the molecular mechanisms that entail this condition, the focus on the determination of the gene variants accountable for hypertension during pregnancy may be enhanced.

## METHODS

This is a case-control study, based on hypertensive pregnant females ministering to the OPD and gynecology ward of Civil Hospital, Karachi. The benchwork was accomplished at Muhammad Ali Jinnah University from 2018 to 2019. Subsequently, board of advance research studies (BASR) of the University of Karachi, Pakistan approved the research. Sample size was calculated by employing the online open epi sample size calculator; the size hence computed was 160 (control:70, hypertensive pregnant females:90) Several subjects were excluded from the study, such as those with a history of diabetes, hyperlipidemia, liver or renal disease, congestive cardiac failure, and recent episodes of myocardial infarction. The control labelled group incorporated normotensive pregnant females whose systolic blood pressure (SBP) readings were < 140 mmHg and diastolic blood pressure (DBP) readings were < 90mmHg. Additionally, they weren’t undergoing any treatment for heart disease or hormone-replacement therapy. The hypertensive group comprised of the pre-diagnosed hypertensive females with SBP > 140 mmHg and DBP > 90 mmHg. A detailed questionnaire regarding the medical history of each subject was recorded, and a thorough physical examination was conducted to ascertain absence of any disorder. The benefits and risks of participation in this study were elucidated verbally to every subject. Furthermore, written consent was obtained from the participants wherein data confidentiality was ensured. The study proceeded by acquiring 5ml of blood and DNA was extracted using Qiagen kit according to manufacturer’s protocol. The TaqMan SNP Genotyping amplification method was utilized to execute the genotyping process in an automated thermal cycler (Agilent Technologies; Sure cycler 8200). A total of 25μl polymerase chain reaction (PCR) reagent mix was prepared using one µl of each primer, 5μl of the DNA sample, 12.5μl of master mix, and rest of the volume was covered by distilled water. It was put through 43 cycles of various steps: denaturation at 95°C for 10 seconds, recombination at 55°C for one minute, and elongation at 72°C for seven minutes. The following predesigned primer pair was used for amplification:^10^

Forward: 5’-TCCCCATCCTTACTGTCTGG-3’

Reverse: 5’-GTTGTGGACTCCGGAACAAT-3’.

Restriction fragment length polymorphism (RFLP) process was used to analyze the PCR products for further genotyping. The amplified products were digested by the restriction enzyme Bsl-1, which were then run on 6% agarose gel in combination with 0.5 ug/ml ethidium bromide at 750 volts for 25 minutes. The results hence obtained were studied and photographed under ultraviolet light using the UVITEC system (Uvitec Cambridge) available in the Bioscience Lab at Muhammed Ali Jinnah University.

Statistical analysis was carried out using the IBM SPSS 23 software. Pearson chi-square test was employed to examine the genotypes and the allele frequency distribution (case/control) and independent t-test was applied for continuous variables. Genotype distribution between the cases and controls was estimated using the Hardy Weinberg equilibrium (HWE). Further, odds ratios (OR) were computed to observe the effects instigated by the differences in alleles and genotypes, at 95% confidence intervals (95% CI).

## RESULTS

We observed significant differences in all baseline characteristics (age; P=0.002, pre BMI; P=0.004, systolic and diastolic blood pressures; <0.001, and gestational weeks; P=0.025) in between healthy and hypertensive pregnant females. Analysis revealed that the patients in our study were over-weight 20.8.7% and obese (I = 20.8.7%; II = 23.8%). On the whole, about 37.8% of hypertensive patients in this study were obese.

**Fig.1:**
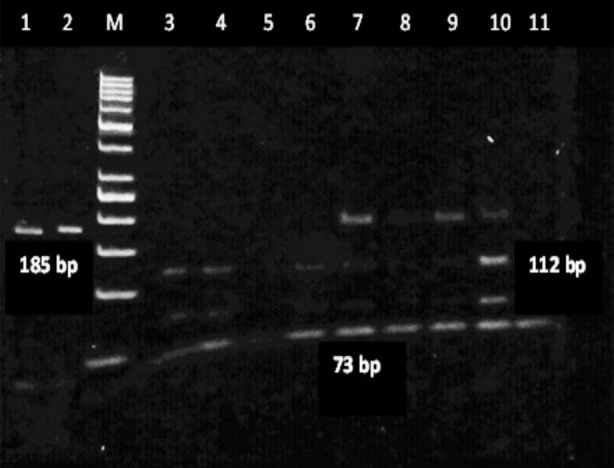
Gel electrophoresis of PCR-RFLP of Prostasin Gene polymorphism in 6% agarose gel.

Overall a PCR product of 185 base pairs (bp) was obtained with prostasin gene amplification. Restriction fragment length (RFLP) analysis was performed, utilizing the Bsl one enzyme. All samples were digested into two fragments of 112 and 73 base pairs (bp). Following electrophoresis on 6% agarose gel, DNA with a single band of 185 bp was considered as TT genotype, two bands of 112 and 73 bp considered as CC genotype, and TC genotype appeared as three bands of 185, 112, and 73 bp (Fig.1).

Genotypic distribution of TT, TC, and CC genotypes between control and hypertensive group is presented in Table-I. A significant difference (P=0.012) was found for the CC genotype, while the TT and TC genotypes were not significantly different (P= 0.06 and 0.49) between the two groups. Moreover, allelic distribution of T and C was calculated as 82% and 18% in control group; 68.2% and 31.8% in hypertensive group, respectively.

**Table-I:** Genotype and Allele Frequencies with Chi square of Prostasin Polymorphism at rs12597511 in Healthy and Hypertensive pregnant females.

SNP Genotype	Control n(%)	Hypertensive n(%)	X^2^	P- values
TT	47(67.2)	47(51.4)	0.463	0.06^ NS^
CC	2(3.1)	13(14.9)	6.31	0.012*
TC	21(29.7)	30(33.8)	3.55	0.49^NS^

SNP: Single Nuecleotide Polymorphism,

* Significant at P<0.05, NS = Non Significant.

Binary regression analysis showed adjusted odds ratios of 6.2 for the CC genotype and 1.33 for the TC genotype when compared to TT. A significant association (P_a_=0.025) was observed for the CC genotype, and a non-significant (P_a_=0.44) for the TC genotype. This confirms that females carrying the CC genotype were 6.2 times more likely to have hypertension and the TC genotype increases the chances of having hypertension by 33%, albeit non-significantly (Table-II).

**Table-II:** Binary logistic regression analysis of risk factors for hypertension during pregnancy.

SNP Genotype or Allele	Control (n)or Mean +SD	Hypertensive (n) or Mean +SD	P_a_ value	AOR	CI (95%)

CC)/TT	2/47	13/47	0.025*	6.2	1.26-30.5
TC/TT	21/47	30/47	0.44^NS^	1.33	0.64-2.7
Pre-BMI (kg/m^2^)	23.4 ± 2.5	25.65 ± 4.0	0.023*	1.05	0.97-1.13
Age	24.61 ± 3.38	26.47 ± 3.98	0.22^ NS^	0.95	0.89-1.03

* Significant at P<0.05,NS = Non Significant

P_a_:adjusted P value, AOR: Adjusted odds ratio and CI: confidence interval.

A Comparison of the preBMI of the subjects showed a significant difference between the two groups (P=0.023) with an adjusted odds ratio (AOR) of 1.05. In other words, each unit increase in preBMI would raise the risk of hypertension by 5% (Table-II).

Regression analysis for allelic frequency presented a significant association of the T and C alleles in control and hypertensive pregnant females (P=0.04). An odds ratio (OR) of 0.48 was observed for the T allele and an odds ratio of 2.08 for the C allele. Since OR for the T allele is less than one, indicates that the T allele is not associated with the disease and has a protective role, whereas, subjects with the C allele are 2.08 times more prone to have hypertension, therefore carrying this allele confers a risk. The genotype distribution between control and hypertensive females suggested that both groups were in the Hardy-Weinburg equilibrium (HWE) because their genotypic frequencies were not significantly different from the expected frequencies.

## DISCUSSION

Present study was conducted to find out polymorphism of prostasin gene at rs12597511 in hypertensive pregnant Pakistani females and to explore the relation of prostasin genotypes with phenotypes. High maternal and fetal morbidity and mortality rates of pregnancy hypertension in developing countries like Pakistan, pose a major burden on health care system. The precise molecular pathophysiology of the disorder remains uncertain, however, our study identified a significantly higher pre-pregnancy body mass index (PreBMI) in hypertensive group as compared to control, which is consistent with the findings of Savitry et al. They stated that higher pre-pregnancy BMI is significantly associated with higher SBP and DBP in hypertensive and preeclamptic patients.^11^ In obese individuals, numerous inflammatory mediators, like C reactive proteins, interleukin 6, and tumour necrosis factor-alpha are released by adipocytes.^12^ All of these substances yield poorer health outcomes and mark obesity as a risk factor of cardiovascular complications; this may also be the cause of having higher prevalence in our study population.

The current research demonstrated that the mean age of patients is closely related to the occurrence of hypertension by having younger females being more in the control group and older in hypertensive group. This indicates an elevated risk of having hypertension with advanced age as reported by many authors in their reviews.^13^-^15^

PreBMI comparison between the control and the hypertensive group showed significant differences (P_a_ = 0.023) with adjusted odd ratio (AOR) of 1.05 after correction of other variables. Similar findings were presented by Savitry et al. who demonstrated that each unit rise in BMI raises the risk of gestational hypertension and preeclampsia by 6% and 9%, respectively.^11^

The study revealed that most of the females were in their 3^rd^ trimester and their mean gestational age was significantly different (P<0.001) between the two groups. However, most of the authors, like Gupta et al. observed no significant difference in the mean gestational age of the patients and control groups. The difference might be because they included only those females having a gestational age of more than 20 weeks.^16^

We observed a strong correlation of CC genotype of prostasin gene (P=0.012) in hypertensive pregnant females with AOR of 6.2, suggesting that pregnant females were 6.2 times more susceptible to have hypertension with this genotype, although TC showed non-significant effects. Moreover, calculation of the allelic distribution indicated a significantly higher prevalence of C allele (P=0.04) in hypertensive group with 2.08 times increase risk of hypertension in pregnancy, however, T allele was not associated with the disease and played a protective role. These results were in agreement with the study performed by Dong et al., however, due to a low frequency of CC genotype, they combined the frequencies of CC and TC together.^17^

In contrast, Zhu et al. demonstrated prostasin polymorphism at rs1549294, rs2855475, and rs12597511 SNPs but only findings pertaining to rs12597511 SNP were statistically significant. Patients with CT and TT genotype had higher BP levels and radial pulse wave velocity (PWV) than those with the CC genotype. Moreover, T allele was described as the minor allele and identified as a risk factor for hypertension.^18^

The incongruence with the results yielded by the present study is likely due to the different ethnicity of the research subjects. Hence it could be inferred from the present analysis, that CC genotype (C allele of rs12597511), could confer a risk factor for developing hypertension in pregnancy. Precise molecular pathways for prostasin in hypertension pathophysiology warrants future research with different ethnicities and broad sample size. In summary, this is the first study conducted in Pakistan, to analyze the effect of variation in the human prostasin gene in hypertensive pregnant females and proposes a novel candidate gene for hypertension. Identifying the causative genes and their variant in developing hypertension is of significant importance and can continue to classify females at risk for potentially life-threatening events during pregnancy.

### Limitations of the study

Firstly, we recruited subjects from a single province. A multi-centre study including subjects from diverse ethnic groups may have provided better insight into the potential role of above polymorphism with pregnancy hypertension. Secondly, information about pre-pregnancy weight was enquired during the first antenatal visit, raising the likelihood of recall bias. It is recommended to conduct multi-centre studies on a large sample size exploring additional polymorphisms via whole genome sequencing.

## CONCLUSION

This study demonstrated a close relationship between rs12597511 prostasin polymorphism and hypertension in Pakistani pregnant females. Women bearing the C allele had a high genetic risk of hypertension. Personalized medicine may be designed to meet the physiological conditions of the patients, depending on their genotype, which can help manage the disease safely and efficiently.

### Authors’ Contributions:

**SE and AA** conceived, designed, interpreted the results, and prepared the draft.

**SR, KA, and AM** did data collection and analysis and critical revision of the manuscript.

**SE and AA** did review and gave final approval of manuscript.
